# IL-17A Induces Endothelial Inflammation in Systemic Sclerosis via the ERK Signaling Pathway

**DOI:** 10.1371/journal.pone.0085032

**Published:** 2013-12-23

**Authors:** Xiaojing Xing, Ji Yang, Xiaoqin Yang, Yi Wei, Lubing Zhu, Di Gao, Ming Li

**Affiliations:** Department of Dermatology, Zhongshan Hospital, Fudan University, Shanghai, China; University of Texas Health Science Center at Houston, United States of America

## Abstract

Recent reports have demonstrated that endothelial cells are involved in vascular inflammatory injury in systemic sclerosis (SSc) and interleukin-17A (IL-17A) plays a crucial role in the pathogenesis of SSC. However, little is known about the effects of IL-17A on endothelial cell inflammation in SSC. The aim of our study was to investigate the role of IL-17A in endothelial inflammation. Here, we showed that IL-17A mRNA and protein levels were augmented in the peripheral blood and more IL-17^+^ lymphocytes infiltrated in the perivascular areas in the involved skin of SSC patients. SSC patient serum induced chemokine and adhesion molecule expression in HUVECs, which was blocked by IL-17A neutralization. IL-17A alone induced chemokine and adhesion molecule expression and promoted T cell-HUVEC adhesion. Extracellular signal-regulated kinase (ERK) inhibition and IL-17A neutralization prominently inhibited chemokine and adhesion molecule expression and blocked T cell-HUVEC adhesion. IL-17A derived from SSC patient serum mediated endothelial cells inflammation by up-regulating chemokines and adhesion molecules, which was blocked by ERK inhibition. These data imply that ERK signal pathway might play a key role in the progression of endothelial injury induced by IL-17A in SSC.

## Introduction

Systemic sclerosis/scleroderma (SSc) is an autoimmune connective tissue disease with unknown etiology and complex pathogenesis. The main manifestations of the disease are cutaneous and internal organ fibrosis. The disease is associated with higher rates of disability and mortality without effective therapy [1]. In the past two decades, advances have been made in understanding the pathogenesis of SSc. It is believed that autoimmunity, inflammation, micro-angiopathy, and fibrosis comprise the quartet of factors contributing to SSc pathogenesis [2]. Accumulating evidence has indicated that vascular injury occurs in the early stage of SSc. Histopathologic evidence of vascular damage has been observed prior to fibrosis and can be detected in both involved and uninvolved skin [3]. The most common initial symptoms and signs are vascular function disorder, like Raynaud phenomenon (RP), which has been often preceded or accompanied skin and internal organ fibrosis by many years [4]. Vascular damage and dysfunction represent the earliest morphological and functional changes of SSc. 

Endothelial cell involvement is the initial and central event in the process of vascular injury in SSc [5]. Immune cell mediates endothelial cell activation, which results in increased expression of proinflammatory factors and vasoactive mediators, such as nitric oxide, prostacyclin, endothelin-1 (ET-1), vascular cell adhesion molecule-1 (VCAM-1), and E-selectin [6]. These inflammatory cytokines activate repair processes and recruit more leukocytes [7]. Inflammation results in platelet and lymphocyte adhesion to the blood vessels, then blood coagulation and fibrinolysis successively occurring [8]. Injured endothelial cells lead to dysfunction, which may result in the irreversible loss of blood vessel integrity [9]. Endothelial cell injury is associated with fibrin deposits, which cause lumen narrowing and vessel obstruction and result in micro-angiopathy formation. Extensive micro-angiopathy results in chronic tissue hypoxia, which promotes and aggravates tissue fibrosis [10]. In addition, the movement of leukocytes and inflammatory cytokines from vessels into the extracellular matrix (ECM) aggravate the fibrosis of the ECM [11].

Accumulated data support a possible role for T helper 17 (Th17) cells in the pathogenesis of SSc, consistent with the role of Th17 cells in other autoimmune diseases [12]. IL-17 is overproduced by T cells in peripheral blood and fibrotic skin and lung lesions in SSc [13]. A cross-sectional study found that the proportion of Th17 cells in peripheral blood was higher in diffuse cutaneous systemic sclerosis than in the limited type [14]. IL-22- and IL-17A-producing T cells with skin- and lung-homing capabilities are characteristically increased in SSc [15]. One study has demonstrated that IL-17A contributes to skin fibrosis in two mouse models of SSc [16]. Although the frequencies of circulating IL-17-secreting T cells have been observed in SSc, however, no clear distinction has been made in these studies between an increasing number of the IL-17-producing T cells and endothelial cells injury in SSc. In the present study, we sought to assess the role of IL-17A in endothelial inflammation in SSc in order to identify a key mediator in the pathogenesis of SSc, which is fundamental to the understanding of the disease.

## Materials and Methods

### Patients with SSc and healthy individuals

Twenty adult patients with a diagnosis of SSc based on the American College of Rheumatology criteria [17] were enrolled in the study after giving informed and written consent. For diagnosis of SSc, we excluded the patients with allergic contact dermatitis and psoriasis regularly based on symptoms, signs and examinations. Blood samples were obtained from the SSc patients. As a control, 16 healthy individuals from Fudan University were enrolled in our study after giving informed consent (Table 1). In this study, the human study protocol was approved by the Institutional Review Board of Zhongshan Hospital of Fudan University. Disease activity was assessed using the criteria proposed by Valentini et al. [18], in which evaluation of clinical and laboratory factors results in a score ranging from 0 to 10 (0 represents no disease activity, and 10 represents maximal activity). The enrolled patients were all active-stage with scores ≥3, and the disease durations were all <5 years. In the group of patients with SSc, 10 patients were receiving hydroxychloroquine plus prednisone, 5 patients were being treated with prednisone plus cyclophosphamide, 3 patients were receiving prednisone alone, 2 patients were not being treated at the time of analysis, and no patients were received with vasodilatory therapies.

**Table 1:** Clinical characteristics of SSc patients included in the study.

Characteristics	SSc	Controls
Total	20	16
Female sex	15	13
Male sex	5	3
Mean age upon inclusion in the study, years (±SD)	42.4±9.2	35.2±7.3
Sclerodactyly	20	
Raynaud phenomenon	20	
Digital ulcers	4	
Esophareal	6	
Pulmonary hypertension	2	
Scleroderma renal crisis	0	
Auto-antibodies scl-70 (+)	9	
Auto-antibodies nticentromere (+)	10	

Skin tissue obtained from skin biopsy specimens was obtained from two patients with SSc who had no treatments. Two healthy tissue specimens were obtained from individuals undergoing orthopedic surgery who provided informed consent. The study protocol was reviewed and approved by the Zhongshan Hospital Research Ethics Committee.

### Immunohistochemical staining of IL-17A

For immunohistochemical analysis, paraffin-embedded sections were obtained with a rotary microtome (Leica, German). Briefly, paraffin-embedded sections were deparaffinized, hydrated, and washed with PBS. After microwave antigen retrieval, endogenous peroxidase activity was blocked by incubation of the slides in 3% H_2_O_2_, and non-specific binding sites were blocked with non-immune serum (Beyotime Institute of Biotechnology, Shanghai, China). Following incubation with primary antibodies, including goat anti-human IL-17A at a concentration of 5 μg/ml (R&D Systems, Minneapolis, MN, USA), at 4°C overnight, the sections were serially incubated with the secondary antibody and streptavidin/peroxidase (SP) (Zhongshan Gold Bridge Biotechnology, Beijing, China). The sections were developed with a DAB Horseradish Peroxidase Color Development Kit (Beyotime Institute of Biotechnology) and counterstained with hematoxylin.

### Detection of IL-17A in serum of SSc patients by ELISA

The concentration of IL-17A in the sera of SSc patients was detected by ELISA according the manufacturer’s instructions. Briefly, recombinant human IL-17A (R&D Systems) was used for standard curves. Standard protein samples and patient plasma samples were added and incubated for 2 hours at room temperature. After washing the samples four to six times, each well was incubated for 1 hour with 50 μl of primary anti-human IL-17A antibody (R&D Systems). After washing as before, 100 μl of enzyme-labeled antibody was added to each well and incubated for 1 hour at room temperature. After washing as before, solutions A and B (R&D Systems) were mixed 1:1 and added to each well. Half an hour later, the reaction was stopped by the addition of 50 μl of stop buffer. IL-17A concentration data obtained from the ELISA are presented as the mean ± SD. The assay was performed in triplicate according to the manufacturer’s recommendations.

### Isolation and culture of PBMCs

 PBMCs of SSc patients and healthy controls were isolated by centrifugation over Ficoll (GE Healthcare, Uppsala, Sweden) and cultured in RPMI-1640 (Gibco, Grand Island, NY, USA) supplemented with 300 mg L-glutamine, 100 units/ml penicillin, 100 units/ml streptomycin (Sigma-Aldrich, St. Louis, MO, USA), and 10% fetal bovine serum (FBS) (Gibco) for 24 hours at 37°C in 5% CO_2_.

### Cell Culture and Treatment

Human umbilical vein endothelial cells (HUVEC) (Promocell, Heidelberg, Germany) were cultured in endothelial cell growth medium (Promocell). The human microvascular endothelial cell (HMEC)-1 cell line (kindly provided by Dr. Lang Yang from the Third Military Medical University, Chongqing, China) was cultured in endothelial cell growth medium MV (Promocell) [19]. Jurkat cells were purchased from the cell bank of the Chinese Academy of Science and cultured in RPMI-1640 medium supplemented with 10% FBS (Gibco). All cells were cultured at 37°C with 5% CO_2_ and 100% humidity. 

To explore the effect of IL-17A on endothelial cells, the endothelial cells were seeded in 6-well plates at a density of 2×10^5^ cells/well and cultured overnight. Then, recombinant Human IL-17A (eBioscience, San Diego, CA, USA) was added in the medium at final concentrations of 20 ng/ml, 10 ng/ml, 5 ng/ml, or 0 ng/ml. The cells were incubated with IL-17A for 12 hours for real-time RT-PCR analysis and 24 hours for western blot analysis. 

To determine whether IL-17A derived from serum of SSc patients induced the endothelial cells damaged. The cells were cultured in the endothelial cell growth medium MV containing 10% serum from SSc patients with or without IL-17A neutralized antibody (4μg/ml) and healthy controls for 24h. The expression of vascular cell adhesion molecule 1(VCAM-1), intercellular adhesion molecule 1(ICAM-1), chemokine (C-X-C motif) receptor 4 (CXCR-4) and chemokine (C-C motif) ligand 20 (CCL-20) were detected by western blot analysis. 

 For some experiments, the endothelial cells were seeded in plates at 90%-100% confluence and then exposed with IL-17A (20ng/ml) for 0min, 10min, 20min and 30min. The protein of the cells was collected, the phosphorylation status of the four major protein kinases (ERK1/2, AKT, P38 and PI3K) were surveyed by western blot. Meanwhile, the endothelial cells were cultured in the endothelial cell growth medium MV containing 10% serum from SSc patients with or without IL-17A neutralized antibody (4μg/ml) and healthy controls for 20min. Then the phosphorylation of the potential protein kinase were surveyed by western blot.

PD98059 (Selleckchem, Houston, TX, USA), a specific inhibitor of ERK1/2 phosphorylation, and SB203580 (Selleckchem), a specific inhibitor of p38-MAP kinase phosphorylation, were used to identify the downstream signaling mediators of the IL-17R. Both PD98059 and SB203580 were dissolved in dimethylsulfoxide (DMSO) (Invitrogen, Grand Island, NY, USA) at a concentration of 10 mM and added to fresh culture medium to achieve the desired final concentrations of 1 μM/ml, 5 μM/ml, and 10 μM/ml. Cells were pretreated with PD98059 or SB203580 for 2 hours, and the incubated with IL-17A at a dose of 20 ng/ml, 10% serum from SSc patients or 10% serum from the healthy controls for 24 h.

### HUVEC and T cell co-culture

HUVECs were seeded into 6-well plates at a density of 1×10^4^ cells/well. HUVECs were pre-stimulated with IL-17A (20 ng/ml) or serum from SSc patients for 24 hours, then co-cultured with Jurkat cells or PBMCs from SSc patients at a 1:5 ratio in the presence of absence of ERK1/2 signaling pathway inhibitor-PD98059 (10 μM/ml) or IL-17A neutralized antibody (R&D Systems) (4 μg/ml) for additional 24 hours. Finally, non-adhered T cells were washed twice with PBS. Five independent microscopic fields were selected randomly for each sample to ensure representativeness and homogeneity to enumerate the T cells that were adhered to HUVECs. 

### Western immunoblotting

Proteins of HUVEC and HMEC-1 cells were extracted using RIPA lysis buffer (Beyotime Institute of Biotechnology) with the protease inhibitor phenylmethanesulfonyl fluoride (Beyotime Institute of Biotechnology). Proteins were separated on 8% or 10% Tris-glycine gels by SDS-PAGE (Beyotime Institute of Biotechnology) and then transferred onto PVDF membranes (Millipore, Billerica, MA, USA). After being blocked with 5% milk for 2 hours at room temperature, the membranes were incubated with primary antibodies, including rabbit anti-human VCAM-1 (1:500, Cell Signaling Technology), rabbit anti-human ICAM-1 (1:1000, Cell Signaling Technology), rabbit anti-human CCL-20 (1:1000, Cell Signaling Technology), rabbit anti-human CXCR-4 (1:1000, Abcam, USA), rabbit anti-human p38 (1:1000, Cell Signaling Technology), rabbit anti-human phospho-p38 MAPK (1:1000, Cell Signaling Technology), rabbit anti-human JNK (1:1000, Beyotime Institute of Biotechnology), rabbit anti-human phospho-JNK (1:1000, Beyotime Institute of Biotechnology), rabbit anti-human ERK1/2 (1:1000, Cell Signaling Technology), rabbit anti-human phospho-ERK1/2 (1:1000, Cell Signaling Technology), rabbit anti-human phospho-AKT (1:1000, Beyotime Institute of Biotechnology), rabbit anti-human AKT (1:1000, Beyotime Institute of Biotechnology), and rabbit anti-human GAPDH (1:1000, Cell Signaling Technology) overnight at 4°C. Membranes were then washed and incubated with appropriate HRP-conjugated secondary antibodies for 1.5 h at room temperature. Proteins were detected with ECL detection reagents (Beyotime Institute of Biotechnology). 

### RNA extraction and real-time RT-PCR

Total RNA of PBMCs of SSc patients, HUVECs and HMEC-1 were extracted with TRIzol reagent (Invitrogen). Complementary DNA (cDNA) samples were synthesized by using the First Strand cDNA Synthesis Kit and oligo(dT) primers (Thermo Fisher Scientific, Waltham, MA, USA). Levels of mRNA for particular genes were examined using SYBR Green PCR Master Mix (TakaRa, Otsu, Japan). The sequences of each primer pair and the product size are presented in Table 2. The expression level of each gene was determined by the 2^-ΔΔCt^ method. Results were normalized against GAPDH. Real-time PCR was performed in triplicate, including non-template controls.

**Table 2:** Primer pairs used for analysis.

Gene	Forward (5’–3’)	Reverse (5’–3’)
GAPDH	AAGGTGAAGGTCGGAGTCAAC	GGGGTCATTGATGGCAACAATA
IL-17A	CCTCAAGTTCCACTT	CACCAGCATCTTCTCCAC
ICAM-1	ATGCCCAGACATCTGTGTCC	GGGGTCTCTATGCCCAACAA
VCAM-1	GGGAAGATGGTCGTGATCCTT	TCTGGGGTGGTCTCGATTTTA
CXCR-4	ACTACACCGAGGAAATGGGCT	TTCTTCACGGAAACAGGGTTC
CCL-20	AGCACTCCCAAAGAACTGGG	CAGAGGTGGAGTAGCAGCAC

GAPDH= glyceraldehyde-3-phosphate dehydrogenase; IL-17 =interleukin-17; ICAM-1 = intercellular adhesion molecule 1; VCAM-1=vascular cell adhesion molecule 1; CXCR-4= chemokine (C-X-C motif) receptor 4; CCL-20= chemokine (C-C motif) ligand 20.

### Statistical analysis

Quantitative data are expressed as the mean ± SD. Statistics were performed using SPSS 13.0 statistical software (SPSS Inc., Chicago, IL, USA). Statistical significance was determined by analysis of variance followed by the Student’s t-test for comparisons of multiple means. Results with P values less than 0.05 were considered statistically significant. 

## Results

### Increased expression of IL-17A in SSc patients

**Figure 1:**
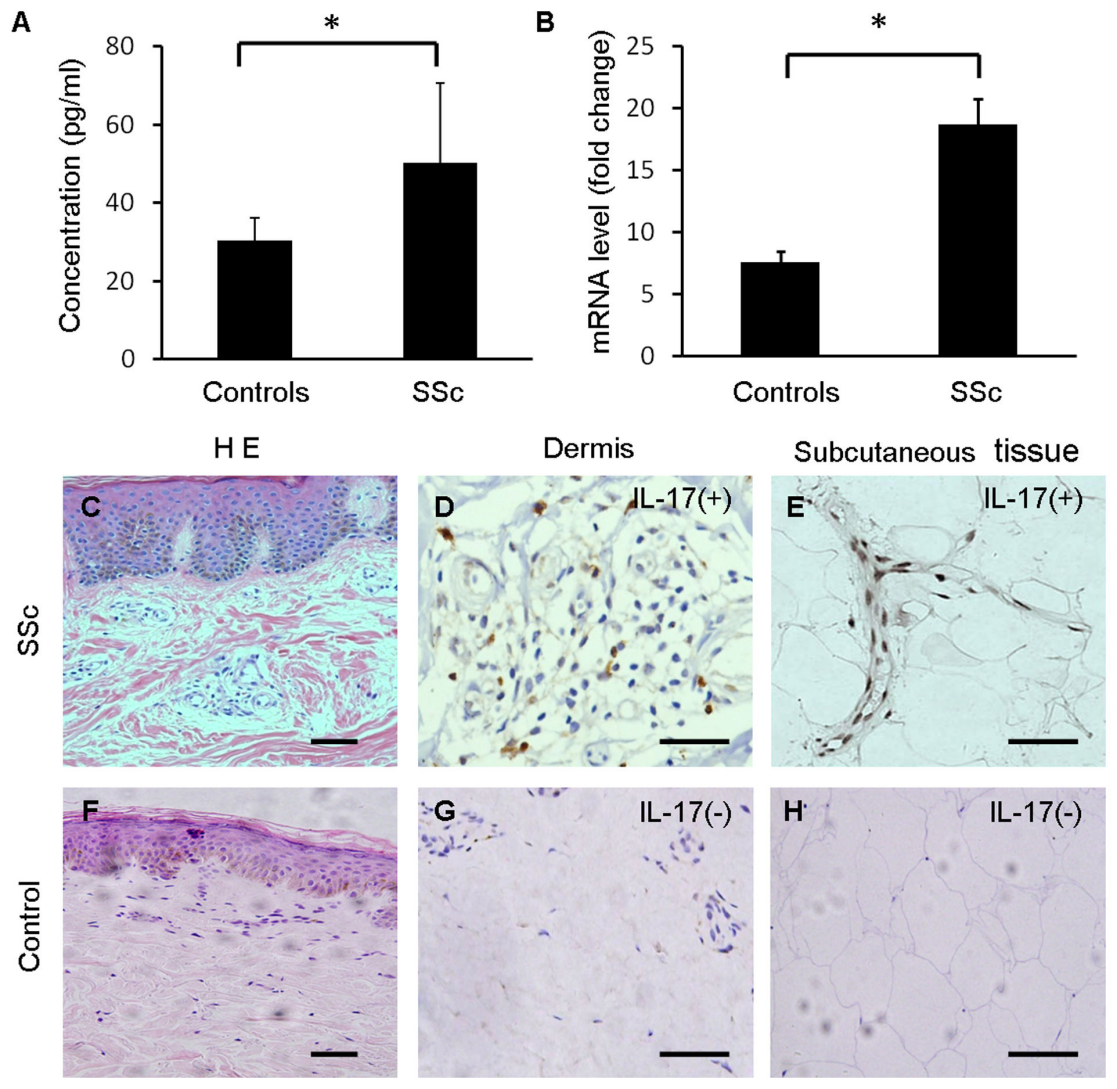
Increased expression of IL-17 in SSc patients. (A) The concentration of IL-17A in the sera of SSc patients (n=20) and healthy controls (n=16) was detected by ELISA. The ELISAs were repeated three times, and the results represent the mean ± S.D. (*P<0.05). (B) Total RNA from PBMCs in SSc patients (n=20) and healthy controls (n=16) was extracted, and real-time RT-PCR analysis was performed for IL-17A. The results of real-time RT-PCR were averaged from three separate experiments (n = 3) and are presented as relative gene expression ±S.D. (*P<0.05). (C) H&E stain of skin in SSc. (D) Expression of IL-17A was detected by immunohistochemistry in the dermis layer of SSc patients. (E) Expression of IL-17A was detected by immunohistochemistry in the subcutaneous tissue of SSc patients. (F) H&E stain of skin in healthy controls. (G) Expression of IL-17A was detected by immunohistochemistry in the dermis layer of healthy controls. (H) The expression of IL-17A was detected by immunohistochemistry in subcutaneous tissue of healthy controls. Arrows show typical positive cells. Scale bars =100 μm.

The gene expression of IL-17A in the PBMCs of SSc patients was detected by real-time RT-PCR, and IL-17A secretion in serum was analyzed by ELISA. We found increased serum concentrations of IL-17A in SSc patients relative to healthy individuals (1.6-fold increase). The concentrations of IL-17A in the sera of SSc patients reached 50 pg/ml (Figure 1A). In addition, our data showed a significant up-regulation of IL-17A mRNA in the PBMCs of SSc patients compared with healthy individuals (10-fold increase) (Figure 1B). 

By using hematoxylin and eosin (H&E) staining, we observed profound dermal inflammation characterized by perivascular lymphocyte and mononuclear cellular infiltrated, perivascular fibrosis deposition, and loss of vessel integrity (Figure 1C). Upon further study by immunohistochemistry, we observed that IL-17^+^ lymphocytes infiltrated the perivascular region, especially the vascular spread over the dermis (Figure 1D) and the septa of fat lobules of subcutaneous tissue (Figure 1E). In the control group, there was no significant IL-17^+^ lymphocyte infiltration (Figure 1F–H). These results provide evidence that IL-17A may be involved in vascular inflammation in SSc patients.

### IL-17A induces the expression of adhesion molecules and chemokines in HUVECs

**Figure 2:**
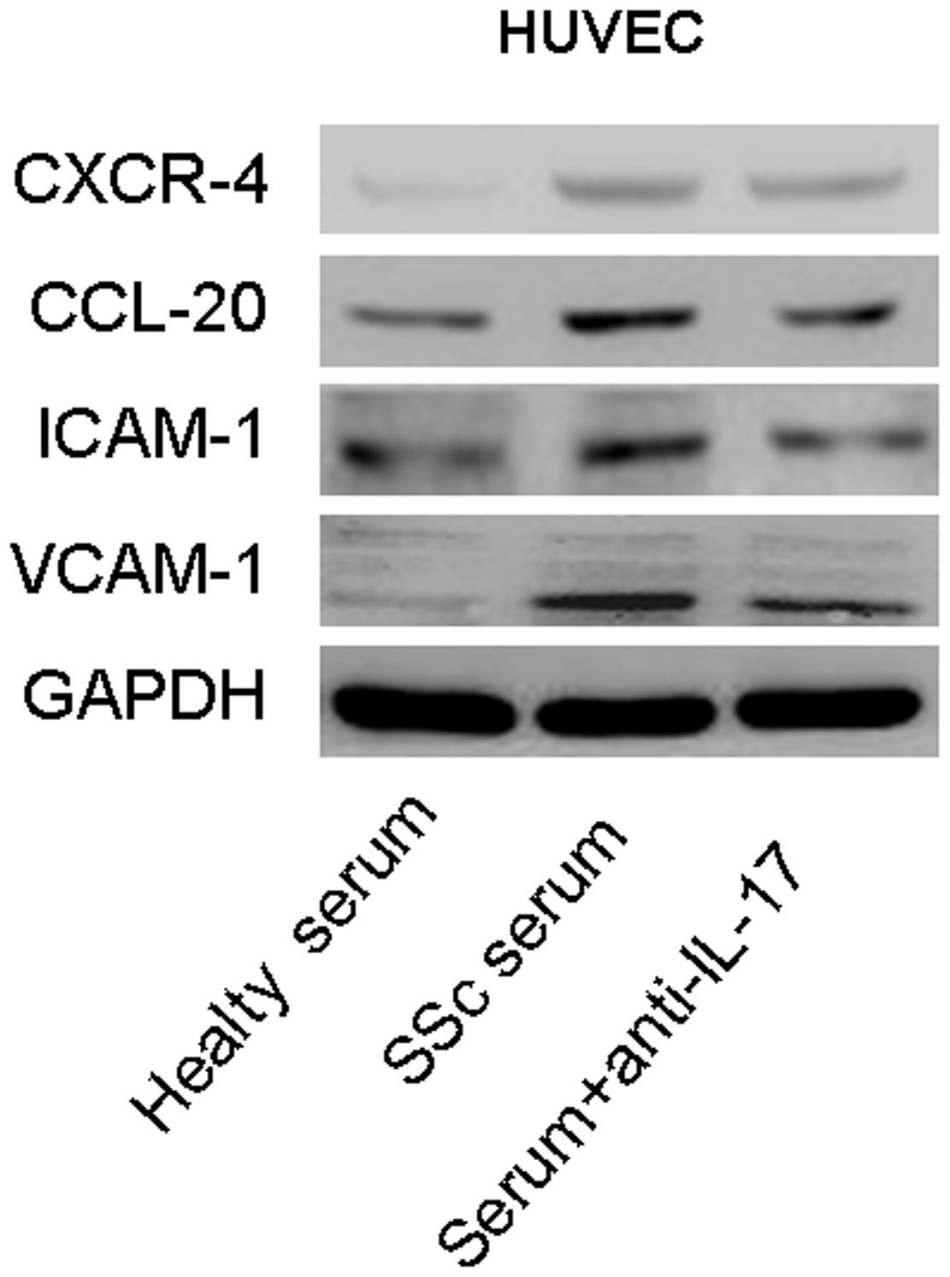
Serum from SSc patients and healthy individuals stimulates the expression of adhesion molecules and chemokines in HUVECs. HUVECs were treated with the serum of SSc patients and healthy individuals for 24 h. Increased expression of CCL-20, CXCR-4, VCAM-1, and ICAM-1 was detected by western blot. The up-regulation of these proteins was partly blocked with IL-17-neutralizing antibody.

**Figure 3:**
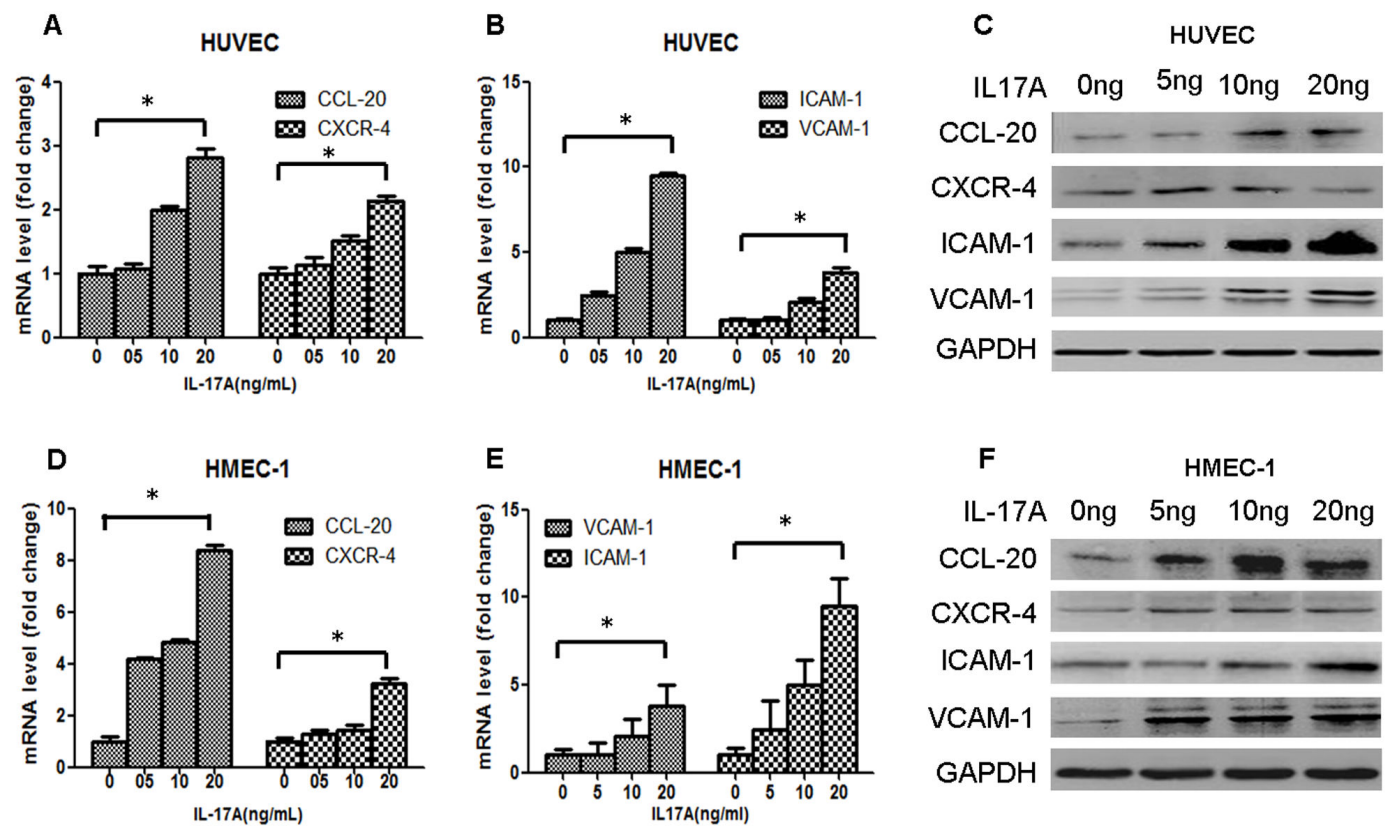
IL-17A promotes the expression of adhesion molecules and chemokines in HUVECs and HMEC-1. (A, D) HUVECs and HMEC-1 were treated with IL-17A at a range of concentrations for 12 h, and mRNA expression of CCL-20 and CXCR-4 was measured by real-time RT-PCR (Comparing 0 ng/ml IL-17A and 20ng/ml IL-17A, *, P<0.05). (B, E) HUVECs and HMEC-1 were treated with IL-17A at a range of concentrations for 12 h, and mRNA expression of adhesion molecules VCAM-1 and ICAM-1 was measured by real-time RT-PCR (Comparing 0 ng/ml IL-17A and 20ng/ml IL-17A, *, P<0.05). (C, F) HUVECs and HMEC-1 were treated with IL-17A at a range of concentrations for 24 h, and protein expression of CCL-20, CXCR-4, VCAM-1, and ICAM-1 was measured by western blot. All experiments were conducted three separate times, and representative data are presented.

We then investigated the role of IL-17A from SSc patients in vascular endothelial cell injury and inflammation. In the IL-17 cytokine family, IL-17A was identified as the main cytokine involved in the pathogenesis of autoimmunity, inflammation, and host defense [20]. Our data show that serum from SSc patients promotes the protein expression of chemokine (C-C motif) ligand 20 (CCL-20), chemokine (C-X-C motif) receptor 4 (CXCR-4), intercellular adhesion molecule 1 (ICAM-1), and vascular cell adhesion molecule 1 (VCAM-1) in HUVECs and that neutralization of IL-17A in culture medium suppressed the expression of these chemokines and adhesion molecules (Figure 2). Furthermore, when IL-17A alone was used to treat HUVECs and HMEC-1, the data proved that IL-17A alone induced mRNA and protein expression of CCL-20, CXCR-4, ICAM-1, and VCAM-1 in HUVECs (Figure 3A-F). Together, these results imply that IL-17A derived from the serum of SSc patients has the potential to induce endothelial cell inflammation.

### IL-17A promotes ERK phosphorylation in HUVECs

**Figure 4:**
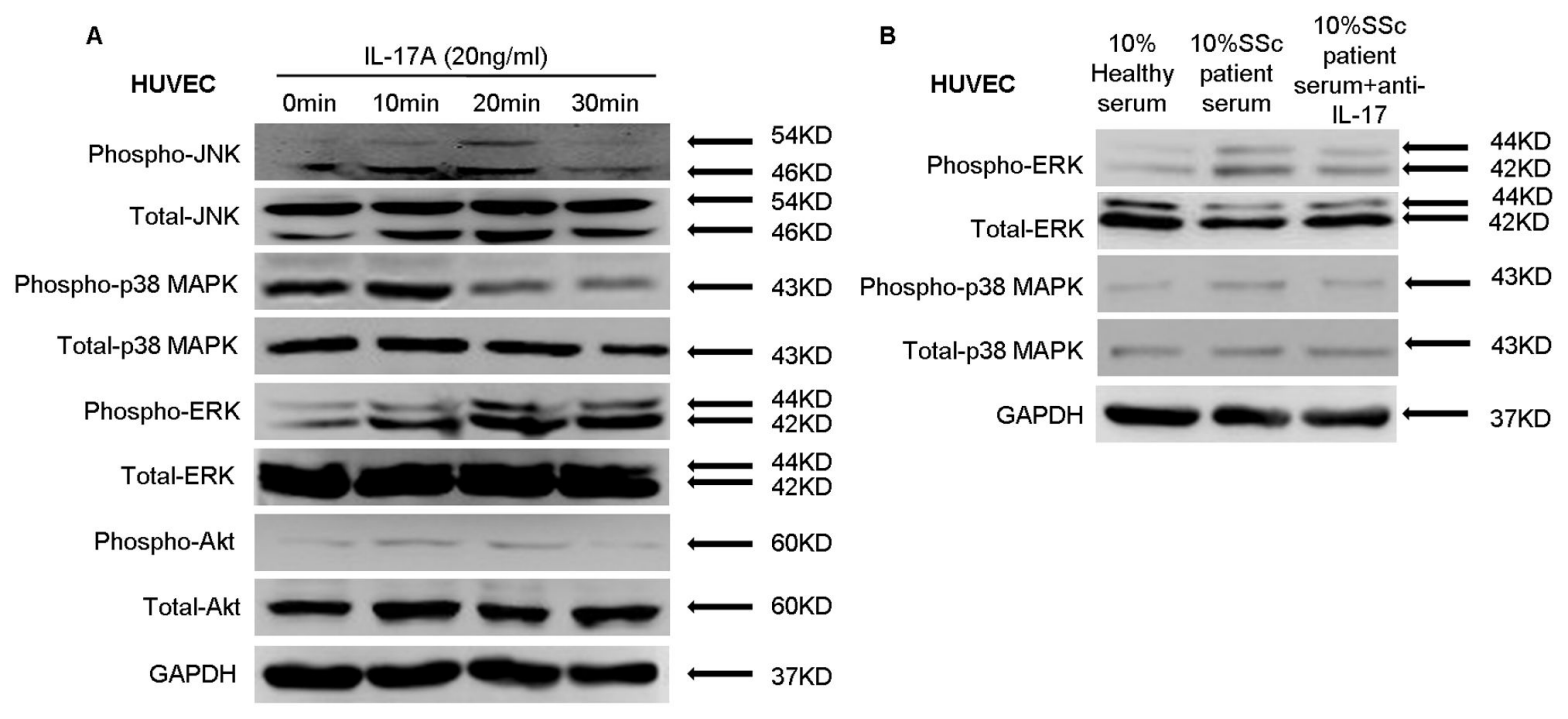
Effects of IL-17A on activation of JNK, p38-MAPK, ERK1/2, and AKT in HUVECs. (A) HUVECs were treated with IL-17A for 10, 20, and 30 min, and the phosphorylation of JNK, p38-MAPK, ERK1/2, and AKT were detected by western blot. (B) HUVECs were exposed to serum from SSc patients and healthy controls for 20 min, the phosphorylation of ERK1/2 and p38 MAPK were detected by western blot. GAPDH was used as a loading control.

Although IL-17A may mediate vascular inflammation, the precise mechanism underlying this effect is still unknown. It is reported that the MAPK signaling pathway may be involved in IL-17-mediated inflammation [21,22]. To determine the potential involvement of protein kinase pathways in IL-17A-mediated inflammation in HUVECs, we evaluated phospho-JNK, phospho-p38 MAPK, phospho-ERK1/2, and phospho-AKT expression in HUVECs by western blot. Prominent increases in phospho-ERK1/2 and phospho-p38 MAPK levels were observed in HUVECs after stimulation with 20 ng/ml IL-17A at 10 min to 30 min. On the contrary, weak expression of phospho-JNK and phospho-AKT were detected at 10 min and 20 min (Figure 4A). In addition, increased expression of phospho-ERK1/2 as well as the phospho-p38 MAPK was detected in HUVECs when exposed to serum from SSc patients. Meanwhile, and the neutralization of IL-17 could weaken the expression of phospho-ERK1/2 and phospho-p38 MAPK (Figure 4B). These experiments revealed that the ERK1/2 and p38 MAPK signaling pathways might be the predominant signaling pathways in IL-17A-mediated vascular inflammation. 

**Figure 5:**
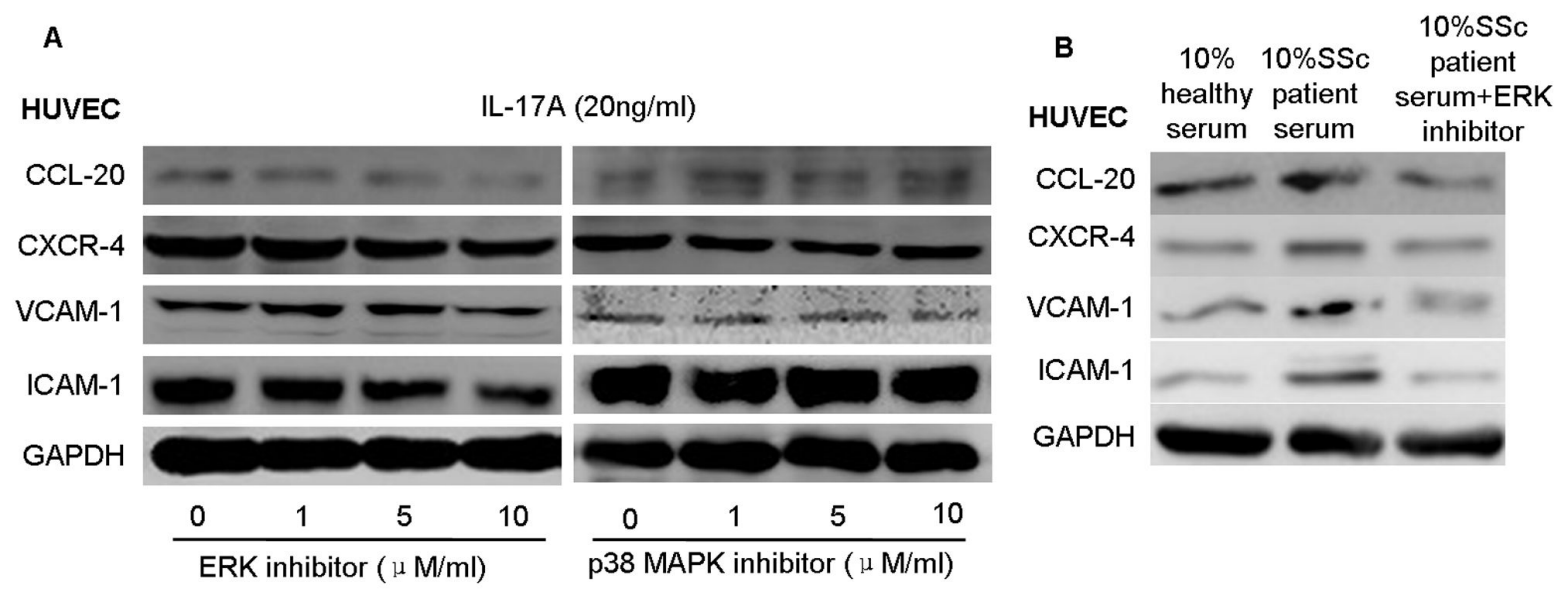
ERK signaling is necessary for IL-17A function in HUVECs. (A) HUVECs were seeded in six-well plates for 12 h and then pre-treated with the different concentrations of PD98059 for 2 h before incubation with IL-17A at 20 ng/ml for 24 h. The expression of chemokines CCL-20 and CXCR-4 and adhesion molecules VCAM-1 and ICAM-1 was detected by western blot. (B) HUVECs were seeded in six-well plates and then pre-treated with PD98059 (10μM/ml) for 2 h, and incubated with serum of SSc patients and healthy controls for 24 h. The expression of chemokines and adhesion molecules were detected by western blot. GAPDH was used as a loading control.

To elucidate the roles of the ERK1/2 and p38 MAPK signaling pathways in endothelial cell inflammation, ERK1/2 inhibitor (PD98059) and p38-MAPK inhibitor (SB203580) were used to treat HUVECs in the presence of IL-17A or serum from SSc patients and healthy controls. PD98059 significantly attenuated IL-17A-mediated up-regulation of adhesion molecules and chemokines in HUVECs. In contrast, SB203580 had little influence on the expression of adhesion molecules and chemokines induced by IL-17A (Figure 5A). Also, the ERK1/2 inhibitor (PD98059) could inhibit the expression of the adhesion molecules and chemokines in HUVECs induced by serum from SSc patients (Figure 5B). These results indicate that the ERK1/2 signaling pathway plays a key role in IL-17A-induced endothelial cell inflammation.

### Blocking ERK activation prevents IL-17A-mediated inflammation reaction in HUVECs

**Figure 6:**
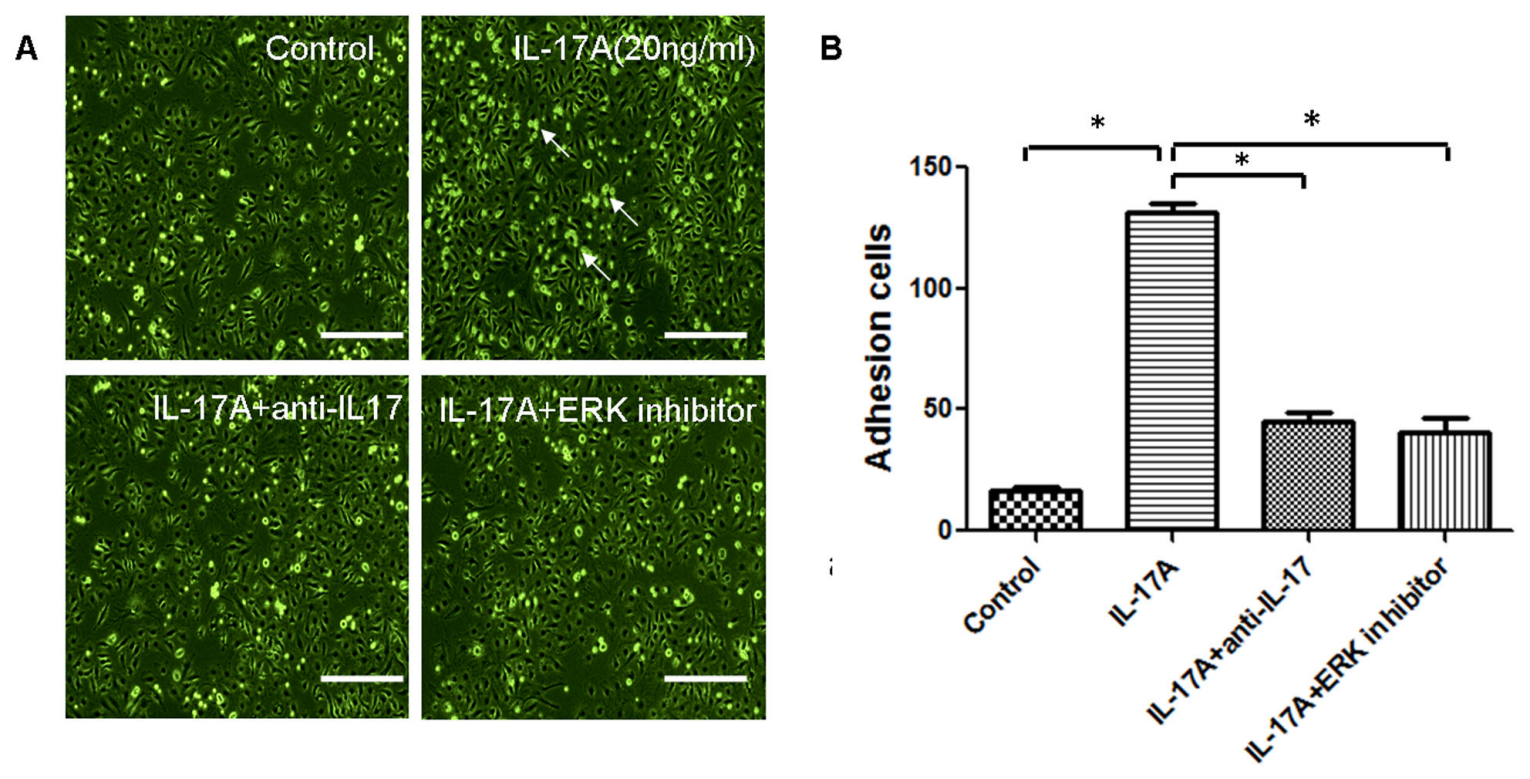
ERK signaling is necessary for IL-17A–induced T cell–HUVEC adherence. (A) Jurkat cells were co-cultured with HUVECs in the presence or absence of IL-17A, IL-17A-neutralizing antibody, or ERK inhibitor (PD98059) for 24 hours, representative picture of Jurkat cells adhering to endothelial cells (Arrows show the adhering Jurkat cells). Scale bar = 100 μm. (B) The number of adherent T cells. The experiment was repeated three times, and the data are presented as mean ± S.D. (*, P<0.05).

To further verify whether ERK activation plays a role in IL-17A–mediated recruitment of T cells to vascular endothelial cells, HUVECs were co-cultured with Jurkat cells. After stimulation with 20 ng/ml IL-17A, a large number of Jurkat cells adhered to HUVECs, and treatment with IL-17-neutralizing antibody or ERK inhibitor significantly reduced Jurkat cell adherence to HUVECs (Figure 6A, B). In addition, ERK inhibitor also prevented the adhesion of PBMCs to HUVECs (Figure S1). These results indicated that IL-17A could promote adhesion of T cells to HUVECs, and ERK1/2 signaling pathway might be involved in IL-17A-induced T cell adherence to HUVECs. 

## Discussion

Accumulating evidence suggests that SSc is a T cell-related disease [23,24]. Helper T cells were observed predominantly around blood vessels in the reticular dermis of SSc patients [25]. However, helper T cells do not represent a homogeneous population, instead containing subpopulations of Th1, Th2, Th9, Th17, and regulatory T (Treg) cells [26]. Among those subpopulations, Th17 cells were found to be strikingly increased in the peripheral blood of SSc patients [27]. Th17 cells were characterized by the secretion of IL-17A, IL-17F, and IL-22 [28]. Our data show that levels of IL-17A are higher in SSc patients than in healthy individuals. These findings are consistent with previous research results [13]. In this study, we observed that IL-17^+^ T cells also infiltrated around the vessels in the dermal and subcutaneous layers. These results suggest that IL-17^+^ T cells might be involved in vascular injury. 

Endothelial cell injury appears to be an initial event in the formation of vasculopathy [29]. Immune elements mediate endothelial cell inflammation, which results in more leukocyte recruitment, adherence to injured endothelial cells, and then leukocyte production of inflammatory mediators to exacerbate the inflammatory reaction and aggravate blood vessel destruction and tissue hypoxia. IL-17A has been reported to mediate the recruitment, activation, and migration of neutrophils [30]. In rheumatoid arthritis, IL-17A may intensify local inflammation by promoting angiogenesis and recruiting innate immune cells into the joints [31]. Other studies have also found that Th17 cells promote the recruitment of additional T cells to vascular endothelial cells in SLE [32,33]. However, the role of Th17 cells in the pathogenesis of vascular injury in SSc remains unclear. Here, we first found that the sera from SSc patients induced the expression of adhesion molecules ICAM-1 and VCAM-1 and chemokines CCL-20 and CXCR-4 in endothelial cells. Furthermore, we showed that this process is suppressed by IL-17A–neutralizing antibody treatment. In addition, we proved that IL-17A, the main factor secreted by Th17 cells, similarly induces the expression of adhesion molecules and chemokines. Moreover, we found that IL-17A promotes T cell adherence to HUVECs. Taken together, these data indicate that IL-17A derived from sera of SSc patients mediates endothelial cell inflammation. 

IL-17A binds the receptor complex IL-17RA–IL-17RC to drive inflammatory gene expression [20]. IL-17A signaling has been shown to activate p38-MAPK signaling [34] and promote primary bronchial epithelial cell growth through the ERK1/2—but not p38-MAPK or JNK—signaling pathway [35]. Treatment of the human gastric adenocarcinoma cell line AGS with IL-17 caused activation of MAPK signaling (ERK, p38, and JNK) [36]. However, the role of IL-17 in endothelial cells remains unclear. In this study, we found that treatment of HUVECs with IL-17A results in increased phosphorylation of ERK1/2 and p38-MAPK but not JNK or PI3K/AKT. In addition, ERK1/2 inhibitor (PD98059) but not p38-MAPK inhibitor (SB203580) significantly inhibited IL-17A–induced expression of VCAM-1, ICAM-1, CCL-20, and CXCR-4. The ERK1/2 inhibitor could further inhibit IL-17-mediated Jurkat cell adherence to HUVECs, which was consistent with a previous study [37]. These data suggest that ERK1/2 phosphorylation is a critical signaling event in IL-17A–induced endothelial inflammation. 

In the pathogenesis of SSc, the damaged microvessel endothelium and surrounding tissue initiate the release of pro-inflammatory mediators, adhesion molecules, and chemokines, which may further promote the recruitment of lymphocytes into the inflamed tissues. The accumulated leukocytes promote inflammation and worsen tissue injury [7]. Here, we demonstrate that IL-17A is over-produced in the peripheral blood and involved skin of SSc patients. IL-17A derived from the sera of SSc patients promotes vascular inflammation by inducing the expression of adhesion molecules and chemokines in endothelial cells in an ERK-dependent manner. 

Currently, we can not draw conclusions that increasing IL-17^+^ lymphocytes infiltrated the perivascular region as a function of micro-angiopathy flare. Regardless of whether this is a cause or a consequence, the expansion of IL-17^+^ lymphocytes is related to distinct cytokine environments in SSc that influence the extent of vascular damage. IL-17 derived from SSc patients serum caused or aggravated the endothelial injury. ERK 1/2 cell signal pathway might play a key role in the progression of endothelial injury indcued by IL-17A in SSc. However, IL-17^+^ lymphocytes was only one of inflammatory cells participated in the vascular injury in SSc, more other inflammatory cells should be analyzed in the pathogenesis of SSc in the future study. ERK cell signal pathway was also one of the cell signal pathways involved in vascular injury induced by IL-17, further study should be done to clarify the complex signal pathway in the pathogenesis of vascular injury in SSc.

## Supporting Information

Figure S1 **The function of ERK 1/2 inhibitor for PBMCs adhering to HUVECs.** (A) PBMCs from SSc patients were co-cultured with HUVECs in the presence of serum from healthy control or SSc patients with or without IL-17A-neutralizing antibody or ERK inhibitor (PD98059) for 24 hours, representative picture of PBMCs adhering to endothelial cells (Arrows show the adhering PBMCs). Scale bar = 100 μm. (B) The number of adherent PBMCs. The experiment was repeated three times, and the data are presented as mean ± S.D. (*, P<0.05).
(TIF)
