# Supplementary figures and images for: IL-17A Induces Endothelial Inflammation in Systemic Sclerosis via the ERK Signaling Pathway
Source: PLoS One. 2013 Dec 23;8(12):e85032. doi: 10.1371/journal.pone.0085032 (PMC3871633; doi:10.1371/journal.pone.0085032)

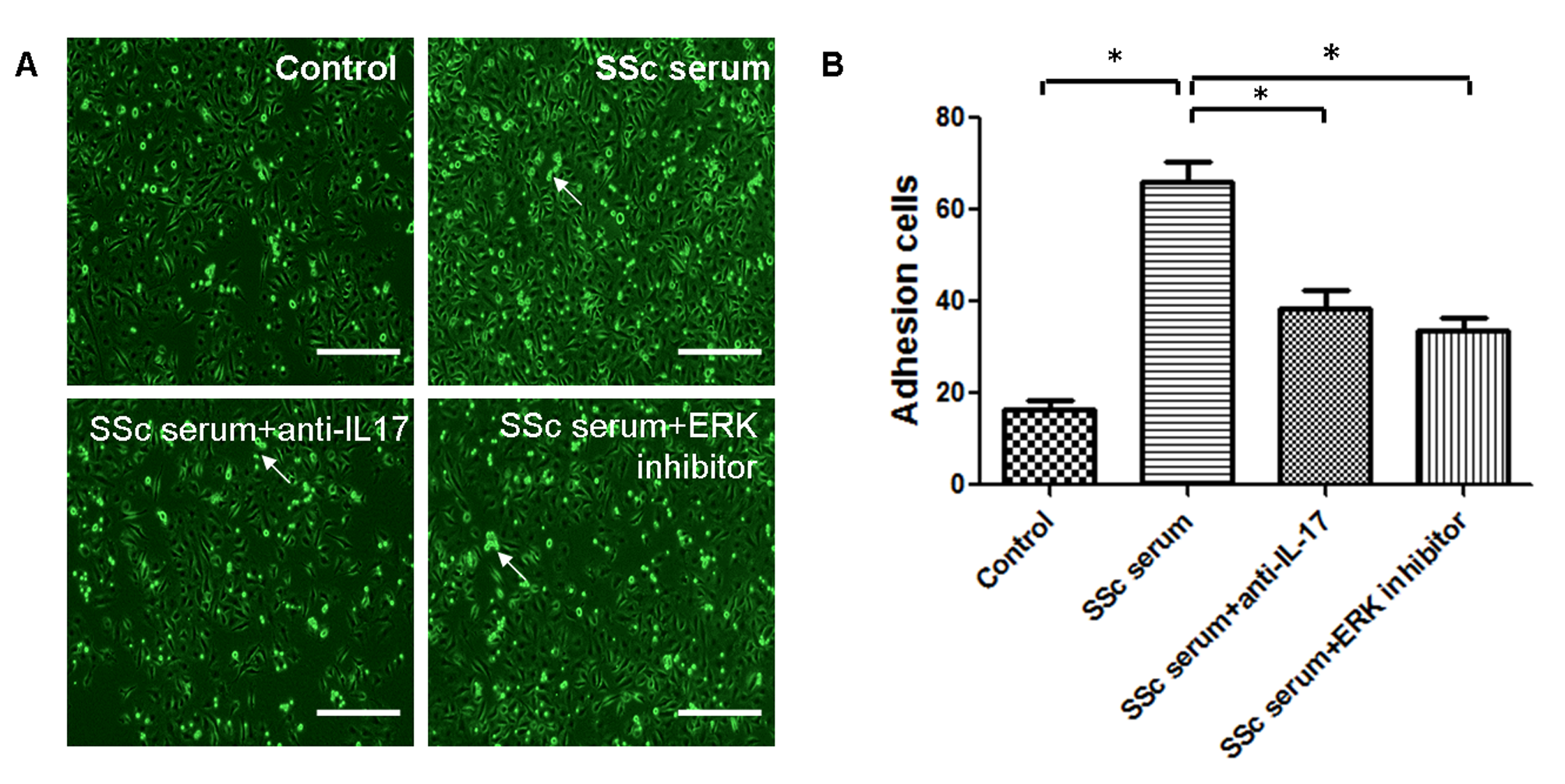

Supplement: Figure S1 — The function of ERK 1/2 inhibitor for PBMCs adhering to HUVECs. (A) PBMCs from SSc patients were co-cultured with HUVECs in the presence of serum from healthy control or SSc patients with or without IL-17A-neutralizing antibody or ERK inhibitor (PD98059) for 24 hours, representative picture of PBMCs adhering to endothelial cells (Arrows show the adhering PBMCs). Scale bar = 100 μm. (B) The number of adherent PBMCs. The experiment was repeated three times, and the data are presented as mean ± S.D. (*, P<0.05). (TIF) [file pone.0085032.s001.tif]
